# Evaluation of COVID-19 intervention policies in South Korea using the stochastic individual-based model

**DOI:** 10.1038/s41598-023-46277-8

**Published:** 2023-11-02

**Authors:** Min-Kyung Chae, Dong-Uk Hwang, Kyeongah Nah, Woo-Sik Son

**Affiliations:** 1 Research Team for Transmission Dynamics of Infectious Diseases, National Institute for Mathematical Sciences, Daejeon, 34047 Republic of Korea; 2 Busan Center for Medical Mathematics, National Institute for Mathematical Sciences, Busan, 49241 Republic of Korea

**Keywords:** Epidemiology, Computational models

## Abstract

The COVID-19 pandemic has swept the globe, and countries have responded with various intervention policies to prevent its spread. In this study, we aim to analyze the effectiveness of intervention policies implemented in South Korea. We use a stochastic individual-based model (IBM) with a synthetic population to simulate the spread of COVID-19. Using statistical data, we make the synthetic population and assign sociodemographic attributes to each individual. Individuals go about their daily lives based on their assigned characteristics, and encountering infectors in their daily lives stochastically determines whether they are infected. We reproduce the transmission of COVID-19 using the IBM simulation from November 2020 to February 2021 when three phases of increasingly stringent intervention policies were implemented, and then assess their effectiveness. Additionally, we predict how the spread of infection would have been different if these policies had been implemented in January 2022. This study offers valuable insights into the effectiveness of intervention policies in South Korea, which can assist policymakers and public health officials in their decision-making process.

## Introduction

Countries have implemented various intervention policies to arrest the spread of COVID-19. Especially in the early days of the pandemic, non-pharmaceutical interventions (NPIs) were implemented due to the absence of a vaccine and a lack of effective treatments^1^. NPIs implemented early in the pandemic included the following; school closures, telework, social distancing, quarantine, and isolation. These intervention policies have effectively slowed the spread of COVID-19, reducing the number of new confirmed cases and admissions to an intensive care unit (ICU)^2,3^.

NPIs are critical for responding to emerging infectious diseases without treatment or vaccine, and the quantitative evaluation of NPIs is essential. For this, we must consider the heterogeneity of populations, including individual demographic attributes such as household members, educational and economic status. Therefore, an individual-based model (IBM) is appropriate for quantitatively evaluating NPIs compared to compartment models, which assume uniform mixing of homogeneous populations. Also, the IBM simulates the transmission dynamics of infectious diseases by considering all contacts with other individuals at home, school, workplace, and in the broader community^4–8^. We can precisely track the infector-infectee tree pairs by recording all contacts where contagion occurs in the IBM. It allows us to calculate a reproductive number by counting the number of infectees per infector^9^ or per day^10^ and measure the effectiveness of NPIs by the change in the reproductive number.

Recently, IBMs have been used to predict the spread of COVID-19 and analyze the effectiveness of intervention policies. In the UK^11,12^, France^13^, Austria^14^, and Singapore^15^, IBMs have been used to study the efficacy of intervention policies in reducing the spread of COVID-19 in each country. The effect of digital contact tracing using the IBM has been investigated^16^. Also, an IBM simulator available in a web app has been released, which can be used by public health officials^17^. Despite these works, there aren’t studies targeting intervention policy of South Korea using the IBM. To analyze the effectiveness of South Korea’s intervention policy, the IBM should be developed according to Korean characteristics. We should design the IBM to allow for population mobility between regions, reflecting the social characteristics of having a high concentration of people in the metropolitan area and the geographical characteristics of being able to travel to any region in a day.

The remainder of this paper is as follows. In “Method” section, we detail our IBM simulation using a synthetic population including various demographic attributes of South Korea and model the transmission of COVID-19. In “Results” section, we present the results of the IBM simulation about November 2020, when stronger NPIs than before were implemented, and evaluate the effectiveness of the intervention policies. We also predict the impact when these policies are implemented in January 2022, and the simulation results with or without intervention policies are compared. In “Discussion” section, we summarize the advantages and limitations of the IBM simulation we developed and describe the potential for future model extensions.

## Method

We develop an individual-based model (IBM) simulation to represent the spread of COVID-19 in South Korea. First, we generate a synthetic population of South Korea using a 2% census and an iterative proportional updating (IPU) algorithm for the IBM simulation. To make the more realistic synthetic population, sociodemographic characteristics are assigned to the synthetic population based on Korean statistical data. The synthetic population has the same size and characteristics as South Korea. The IBM simulation is modeled with an individual’s daily routine based on the characteristics of the individual. The individuals visit multiple places and come into contact with many people in their daily lives, and they can also meet people outside of their residence region. In the simulation, regular contacts (such as households) and irregular contacts (such as a meeting with friends) occur. Finally, we describe the epidemiological parameters and the probability of infection. The epidemiological parameters are adapted from COVID-19 research. The individuals have different infection characteristics. The spread of an infectious disease occurs in a community through person-to-person contact. This simulation permits population movement between regions, which allows for inter-regional transmission of infection. It is stochastically determined whether the susceptible individual is infected when the susceptible individual encounters the infector.

### Synthetic population with sociodemographic characteristics

We make a synthetic population of South Korea to study the spread of COVID-19. The synthetic population is the same size as South Korea and is assigned sociodemographic characteristics. We use the 2015 2% census provided by the MicroData Integrated Service (MDIS) to generate the synthetic population. The 2% census is a population profile of 927,843 people in 382,217 households. The census contains household ID, residence region, and age information. The number of regions is 250.

**Figure 1:**
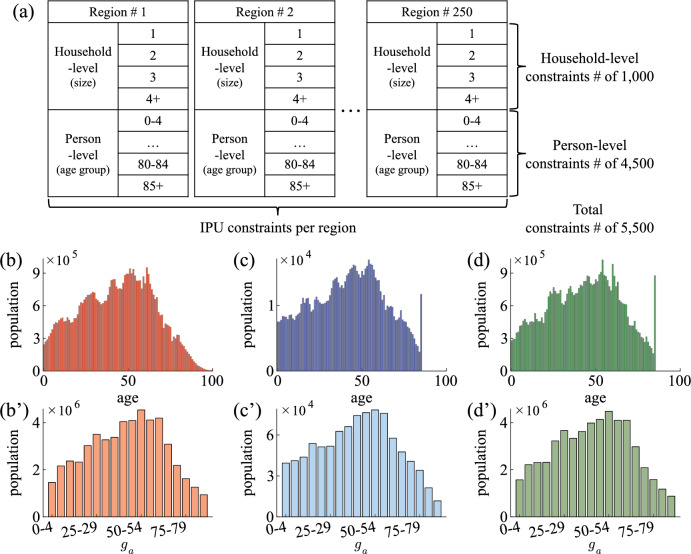
(**a**) Constraints of IPU algorithm; household-level and person-level for the 250 regions of South Korea. The household-level constraint is the number of households by region for households with one to four or more people. The total number of household-level constraints is 1000. The person-level constraint is the number of people by region for age groups in five-year intervals with $85+$ as one group. The total number of person-level constraints is 4500. The total number of constraints in both household- and person-level conditions is 5500. (**b–d**) Age distributions with 1-year intervals and (**b’–d’**) age group $g_a$ distribution with 5-year intervals ($85+$). The distributions of (**b,b’**) South Korea, (**c,c’**) the 2% census, and (**d,d’**) the synthetic population. (**c,d**) The oldest person in the census is 85 years old, so the populations over 85 years old in South Korea are 85 years old in the synthetic population.

An iterative proportional updating (IPU) algorithm is to generate the synthetic population to match household-level and person-level constraints numerically^18^. By applying the IPU algorithm to the 2% census, we can expand the about 920*k* population (2%) to about 51*m* population (100%). In the study, there are two levels of constraints; household-level and person-level (see, Fig. 1a). The household-level constraint is the number of allowed households for given regions and household sizes. The person-level constraint is the number of allowed people for given regions and age groups. These constraints are based on statistical data from the KOrean Statistical Information Service (KOSIS)^19,20^. The number of households in South Korea is 21,448,463, and the population is 51,738,071. The synthetic population has 21,471,466 households and 51,765,522 individuals. The number of households and people in the synthetic population is approximately equivalent to South Korea. We also compare the age distribution as the person-level constraint (see, Fig. 1b–d). Figure 1b’ is identical to Fig. 1d’. We verify that the synthetic population has been successfully generated according to these constraints. This means that the age structure of the generated synthetic population reflects the characteristics of the actual Korean age structure.

**Figure 2:**
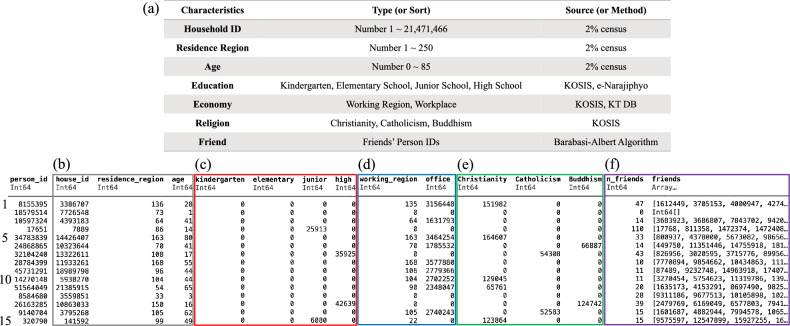
(**a**) Sociodemographic characteristics in the simulation. (**b–f**) Parts of the synthetic population; (**b**) data in the 2% census, (**c**) education, (**d**) economy, (**e**) religion, and (**f**) friend. The value 0 means not applicable. For example, the individual in row 1 belongs to household # 3,386,707, lives in region # 136, and is 28 years old. He doesn’t attend an educational institution but works at workplace # 3,156,448 in region # 135. He visits a Christian religious facility # 151,982 and has 47 friends. The row 4, 7, and 13 are students. The row 15 is a teacher.

An individual has sociodemographic characteristics in the synthetic population of the simulation. We assign sociodemographic characteristics to the generated synthetic population to create a virtual South Korea that is more realistic. There are seven characteristics; household ID, residence region, age, education, economy, religion, and friend (see, Fig. 2a). Three characteristics (household ID, residence region, and age) are information from the census. The others are assigned to individuals based on statistical data from South Korea^21–30^.

Figure 2b–f is part of the synthetic population profile we generated (see, the Supplementary Material for details on how to assign each attribute). The census information is shown in Fig. 2b. The education information shows whether an individual belongs to an educational institution (see, Fig. 2c). Students (ages 3–18) and teachers (ages 19–84) have education information. In the simulation, the education information is a school classroom number where they belong. There are four educational institutions; kindergarten, elementary, junior, and high school. Figure 2d is economy information about whether an individual is economically active or not. The economically active individuals (ages 19-84) are teachers and office workers. They have a workplace number where they belong. For teachers, the school classroom number corresponds to the workplace number. They may also commute to a different region for work, so their working region is also recorded in economy information. Figure 2e is religion information based on whether they are religious or not. There are three religion; Christianity, Catholicism, and Buddhism. They are given the religious facilities number they attend if they are religious. Lastly, we make friends network in the synthetic population. The friend information (ages $3+$) is a list of an individual’s friends (see, Fig. 2f). The list of friends is generated using the Barabasi-Albert network^31^.

### Daily contact routine

The infectious disease spreads through daily contact between the infector and susceptible people in a community. There are many kinds of daily contact. For example, people’s daily routines are as follows; people stay home, go to work/school on weekdays, and often attend a religious facility. Sometimes, people can be in close contact with others through irregular outside activities. People would have irregular get-togethers with their friends. People can also make random contact with strangers, e.g., at the grocery store, on public transportation, etc.

**Figure 3:**
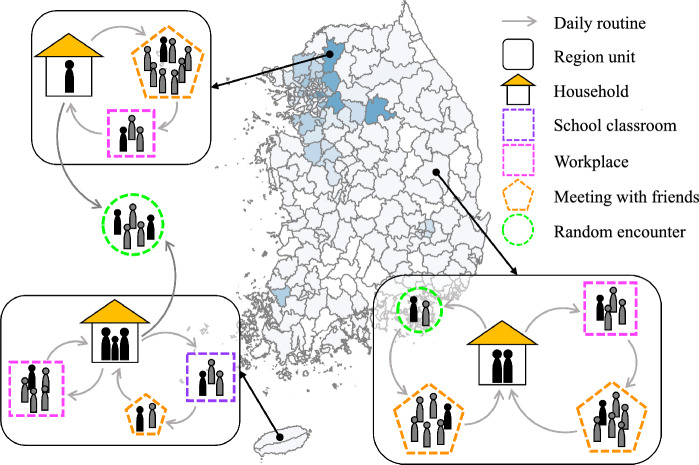
Illustration of contact type in the IBM for a weekday. The all maps of South Korea in the manuscript are created by in-house software by using Observable (www.observablehq.com).

We simulate the daily life of each individual in the synthetic population with sociodemographic characteristics (see, Fig. 3). In the IBM simulation, there are six possible contact types; household, school classroom, workplace, religious facility, meeting with friends, and random encounter. Households, school, workplaces, and religious facilities are places where contacts occur regularly. In the household, contact happens every day. The school and workplace are only visited on weekdays, and the religious facility can be attended on Sundays. The individual’s choice stochastically determines to attend a religious facility, so the contact size of the religious facility varies from week to week. We assume the attendance probability for Christianity, Catholicism, and Buddhism are 80%, 10%, and 10%, respectively^32^. On the other hand, meetings with friends and random encounters occur irregularly. The difference between the two irregular contacts is evident. Meeting with friends is the contact of individuals in an individual’s friends list, while random encounter is the contact with strangers. We embody meeting with friends by creating a “list of friends”, which is one of the sociodemographic characteristics in the synthetic population. The individuals determine whether to meet their friends by their choices every day. We assume that the probability of meeting with friends is 30%. The friends’ meeting size is assumed to be from 2 to 10. The random encounter is an outside activity that involves random contacts. Individuals may have random encounters outside their residence region. They can travel outside of their residence region based daily train and plane ridership data for 2020 between regions provided by the Korea Transform Database (KTDB)^29^. We assume an individual has a 30% probability of random encounters, and the average number of contacted people is 6.

### Epidemiological parameters

**Figure 4:**
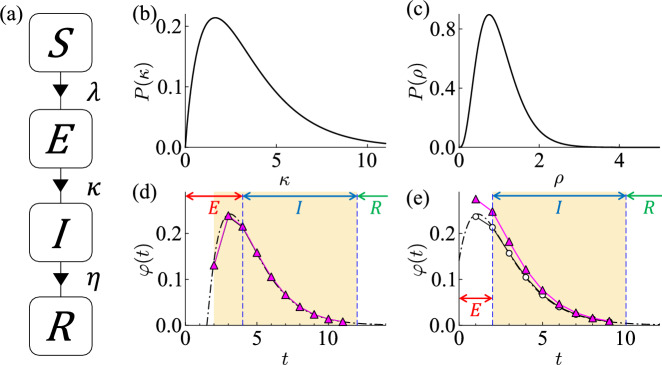
(**a**) Schematic representation of the epidemic model. The progression of an infectious disease is divided into four states; susceptible *S*, exposed *E*, infectious *I*, and recovered *R*. The probability of infection is $\lambda$ ($S \rightarrow E$). After $\kappa$ days in *E*, an individual becomes *I*, then *R* after $\eta$ days. (**b**) Distribution of the incubation period $\kappa$ with a mean of 3.5 days and a standard deviation of 2.3 days. (**c**) Distribution of relative infectiousness $\rho$ with a mean of 1 and a standard deviation of 0.5 ($\left<\rho \right> = 1$ and $\sigma _\rho = 0.5$). (**d,e**) Infectiousness profile $\varphi$ with $\eta =8$ and $\rho = 1$. The black dash-dot line is the viral shedding profile. The magenta triangle symbol is infectiousness, the product of relative infectiousness and normalizing viral shedding. Infectiousness $\varphi$ profile of an infector with (**d**) $\kappa =4$ and (**e**) $\kappa =2$.

We simplify the disease progression into four states; susceptible *S*, exposed *E*, infectious *I*, and recovered *R* (see, Fig. 4a). Initially, the entire population is susceptible *S*. If an individual in *S* comes into contact with an infector. The *S* individual will stochastically become the exposed *E* (in a stochastic manner, $\lambda$). After an incubation period $\kappa$, the *E* individual becomes an infectious *I*. After the duration of the infection $\eta$, the *I* individual gets recovered *R*.

The infectious disease is modeled as COVID-19. Infectors may have the distinct characteristics such as incubation period $\kappa$, duration of infection $\eta$, relative infectiousness $\rho$, viral shedding $\xi$ and infectiousness $\varphi$. The incubation period $\kappa$ and duration of infection $\eta$ are based on the existing literature^33,34^. The incubation period $\kappa$ is different for each individual. The incubation period of an individual is drawn from a gamma distribution with a mean of 3.5 days and a standard deviation of 2.3 days (see, Fig. 4b). The maximum incubation period is assumed to be 10 days. The duration of infection for all individuals is fixed at 8 days ($\eta =8$). The relative infectiousness $\rho$ of an individual is assumed to be picked from a gamma distribution with a mean of 1 and a standard deviation $\sigma _\rho$ (see, Fig. 4c)^4^. The infector spreads an infectious disease to susceptible individuals while actively emitting the virus. The viral shedding $\xi$ of COVID-19 has a gamma distribution with a mean of 3.067 and a standard deviation of 2.109^33^. Infectiousness $\varphi$ is how easily an individual can infect others during the infection. In other words, the individual’s infectiousness is determined by the amount of viral load. Infectiousness $\varphi$ is calculated product of relative infectiousness $\rho$ and viral shedding $\xi$ ($\varphi = \rho \xi$).

In this study, we assume the virus is no longer shed once the infector becomes *R*. The virus is shed 2 days before the end of the incubation period. We also set the virus shedding to start at least 1 day after being infected. The individual can infect others for up to 10 days because the infection lasts 8 days. The viral load is the highest 1 day before *I* and weakens over time. Infectiousness over time of infectors with different incubation periods are shown in Fig. 4d, e. Figure 4d shows infectiousness for an individual with an incubation period of 4 days ($\kappa =4$ where $\eta =8$ and $\rho =1$). The *S* individual became *E* at $t=0$, *I* at $t=4$, and *R* at $t=12$. In this case, the virus is shed for a total of 10 days ($t=2\sim 11$). Figure 4e shows an individual’s infectiousness with an incubation period of 2 days ($\kappa =2$ where $\eta =8$ and $\rho =1$). The *S* individual became *E* at $t=0$, *I* at $t=2$, and *R* at $t=10$. With $\kappa =2$, virus shedding begins 1 day after being infected and 1 day before the end of the incubation period. The virus is shed for a total of 9 days ($t=1\sim 9$). The dashed-dot line with circle empty symbol is viral shedding. The magenta triangle symbol results from normalizing viral shedding to 9 days. The viral shedding is over when the individual is in *R*. They can no longer infect others.

1 $$\begin{aligned}{} & {} \lambda _i = \Sigma ^{6}_{n=1}{\left( \beta _n \frac{\Sigma _j{{\varphi _j \left( t_j \right) }}}{N_n^{\alpha _n}} \right) } \end{aligned}$$

2 $$\begin{aligned}{} & {} \varphi _j \left( t_j \right) = \rho _j \xi \left( t_j \right) \end{aligned}$$

We simulate the epidemic spread by tracking the daily activities of individuals in the synthetic population. We study the stochastic infection spread using this IBM simulation. The susceptible individuals may come into contact with infectors at various places and become infected stochastically. The probability of infection $\lambda _i$ is computed for each individual *i*. Whether infected or not is determined by the state of the population in the places that the susceptible individual visited all day. Motivated by Ferguson^4^, $\lambda _i$ is given by Eq. (1). *n* indexes places ($n\in [1,6]$ where household, school classroom, workplace, religious facility, meeting with friends, and random encounter). $\beta _{n}$ are transmission coefficients for each place. $N_1$, $N_2$, and $N_3$ are the sizes of households, school classrooms, and workplaces. These values are given for each individual *i*. $N_4$, $N_5$, and $N_6$ are the sizes of religious facilities, meetings with friends, and random encounters that individual *i* attended that day. These sizes are not fixed and vary in every situation. $\alpha _1$ is a power determining the scaling of household transmission rates with household size ($\alpha _1=0.8$). Otherwise is 1 ($\alpha _{2} \sim \alpha _{6} = 1$).

The individual *i* is assumed to always start the day at the household. Individuals cannot be in multiple places at the same time. In the simulation, $\lambda _i$ is calculated at the end of an individual’s day ($\textrm{d}t = 1\ \textrm{days}$). If there is an infector *j* in a place that a susceptible individual *i* visited during the day, the individual *i* may become infected through contact with the infector *j*. To get $\lambda _i$, we compute the infectiousness $\varphi _j$ of the infector *j* contacted with the susceptible individual *i* that day (see, Eq. 2). $\varphi _j$ is the product of the viral shedding $\xi$ and the relative infectiousness $\rho _j$ of the infector *j*. $t_j$ indexes the time since the infector *j* was infected. $\lambda _i$ is computed based on the current states of infectors and the contact size an individual *i* met during the day. If a random number from the range $\left[ 0,1\right]$ is less than the $\lambda _i$, then the susceptible individual *i* transit from *S* to *E* (see, Fig. 4a).

## Results

The spread of COVID-19 and intervention policies in South Korea are simulated using our individual-based model (IBM) described above. We reproduce the intervention policies implemented from November 2020 to February 2021 and evaluate their effectiveness, and then predict how the spread of infection would have changed if these policies had been implemented in January 2022, when the number of new confirmed cases increased rapidly.

### Non-pharmaceutical interventions for November 2020

**Figure 5:**
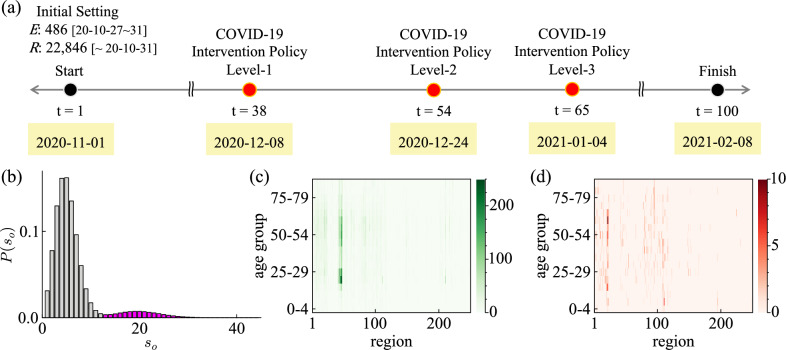
(**a**) Schematic representation of intervention policies for 100 days from November 1, 2020, to February 8, 2021. (**b**) Distribution of the workplace size $s_o$. (**c,d**) Initial setting of the IBM simulation. Heatmaps are ploted by Julia (packages: Plots, LaTeXStrings, Dates). (**c**) Heatmap depicts the recovered population by region and age group through October 31, 2020. (**d**) Heatmap depicts the exposed population by region and age group from October 27 to 31, 2020.

We reproduce South Korea’s intervention policies using the COVID-19 spread model. Among the many intervention policies during the pandemic, we focus on the policies that were implemented from November 2020 to February 2021. The period is relatively early in the pandemic. In the absence of an effective treatment and vaccine, the number of infections started to increase. So, stronger non-pharmaceutical interventions (NPIs) were implemented than before. Three phases of increasingly stringent intervention policies were implemented relatively quickly. Figure 5a shows the timeline of the 100-day intervention policies from November 1, 2020, to February 8, 2021. (1) On December 8, 2020, the COVID-19 Intervention Policy Level-1 was implemented. Level-1 is the intervention policy for schools and workplaces. Schools are encouraged to ease the crowding, and workplaces are encouraged to telework. Reduced crowding means that only 1/3 of each school’s capacity can attend. In the high schools, up to 2/3 of the school’s capacity was allowed to attend. In the simulation, we model the school case by rotating to attend 1/3 of the educational institution capacity per region (2/3 in high schools). However, teachers still go to work every weekday. The telework suggestion is implemented for larger workplaces to randomly attend to only 1/3 of the workplace’s capacity. The distribution of workplace sizes is a double-gaussian (see, Fig. 5b). The right peak is large workplaces ($13+$ individuals). (2) On December 24, 2020, the COVID-19 Intervention Policy Level-2 was implemented. Level-2 prohibits private gatherings of more than 5 people in the national capital area (Seoul, Incheon, and Gyeonggi). In the national capital area, we set the meeting size with friends is limited to a maximum of 4 from 10, and the transmission coefficient for meetings with friends $\beta _5$ is adjusted to $\beta _5/2.5$. (3) On January 4, 2021, the COVID-19 Intervention Policy Level-3 was implemented. Level-3 is that level-2 is expanded to the entire country. We also extend it in the simulation. $\beta _5$ is adjusted to $\beta _5/2.5$ because it is speculated that the spread of the infection may have been further curbed as regulations expanded across the country.

We simulate 100 days from November 1, 2020, to February 8, 2021, to analyze the effects of the three-phase intervention policies. We use the line-level case data in South Korea provided by the Government-Wide R &D Fund for Infections Disease Research (GFID) to set up the initial populations in the immune and exposed state. To simulate November 1, we set the population to be immune to COVID-19 as many confirmed cases as October 31. In addition, people who can shed enough virus to infect others on November 1 have been infected for less than or equal to 5 days (see, Fig. 4d, e), so we assume the population as many confirmed cases from October 26–31 are exposed to COVID-19 on November 1. In other words, the confirmed cases through October 31, 2020, are categorized by region and age group (see, Fig. 5c). We randomly select individuals in the synthetic population for each condition. The chosen individuals are in *R*. They have already been immune at the start of the simulation. The confirmed cases from October 26 to 31 are also divided by region and age group (see, Fig. 5d). We randomly assign *E* in the synthetic population to match the population of each condition. Figure 5c, d show heatmaps depicting the *R* and *E* initial populations.

**Figure 6:**
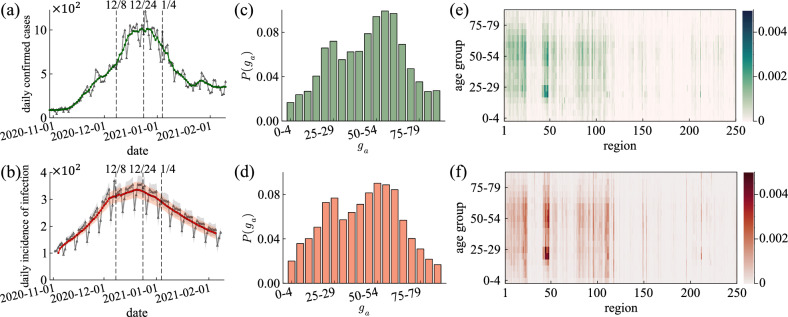
(**a,c,e**) Actual data on COVID-19 in South Korea. (**b,d,f**) Results of the simulation. (**a**) New confirmed cases in South Korea for 100 days from November 1, 2020, to February 8, 2021. The grey line is daily confirmed cases. The green line results from a 7-day moving average on the daily confirmed cases. The dashed lines show the dates when each intervention policy was implemented. (**b**) Result for daily incidence of infection with $\beta _n = 0.8$, $\left<\rho \right>=1$, and $\sigma _\rho = 0.5$ (CI 50%). The grey line is the daily incidence of infection. The red line results from a 7-day moving average on the daily incidence of infection. The dashed lines show the dates when each intervention policy was implemented. (**c,d**) Distribution of infected population by age group $g_a$ through February 8, 2021. (**e,f**) Heatmap depicts infected population by region and age group through February 8, 2021. Heatmaps are ploted by Julia (packages: Plots, LaTeXStrings, Dates).

Figure 6 shows daily new confirmed cases in South Korea and the results of the IBM simulation. Figure 6a, b are the spread of infection over 100 days from November 1, 2020, to February 8, 2021. Figure 6a is the actual daily confirmed cases in South Korea. Figure 6b is the daily incidence of infection (exposed population), which is the result that reproduces Fig. 6a using the IBM simulation [$\beta _n = 0.8$, $\left<\rho \right>=1$, and $\sigma _\rho = 0.5$, 50% confidence interval (CI)]. The total number of simulations is $200+$. Dashed lines indicate the dates when the intervention policies were implemented in Fig. 6a, b. The exact infected date is unknown in the real world, so only daily confirmed cases are recorded. However, in the IBM simulation, the exact infected date can be recorded for each individual, so we observe the daily incidence of infection. We identify that the infection spread is repressed during intervention policies. As the intervention policy is strengthened three times, the infection spread is almost diminished. Figure 6c, d are the distribution of the infected population by age group $g_a$ through February 8, 2021. Figure 6c is plotted using the line-level case data provided by GFID. Figure 6d is the result of the simulation. Figure 6c is identical to Fig. 6d. Figure 6e, f are heatmaps of the infected population by region and age group through February 8, 2021. Figure 6e is the actual data, and Fig. 6f is the simulation results. Figure 6e is identical to Fig. 6f. From these results, we can validate that the simulation reproduces the COVID-19 spread in this period with intervention policies (see, Fig. S8 in the Supplementary Material for a sensitivity analysis of the parameters $\beta _n$ and $\sigma _\rho$).

**Figure 7:**
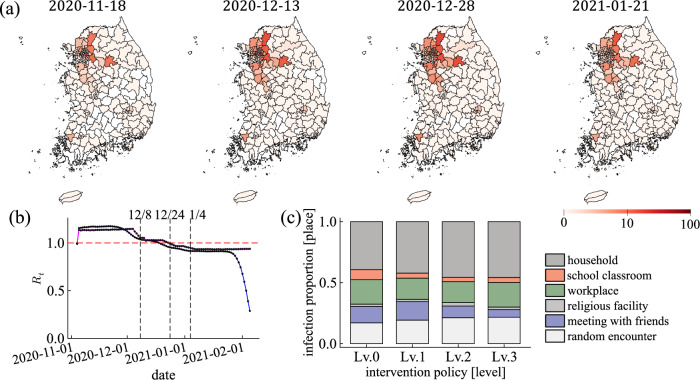
The simulation result with $\beta _n = 0.8$, $\left<\rho \right>=1$, and $\sigma _\rho =0.5$. (**a**) Plots the incidence of infection as a map of 250 regions (Log-Scale). (**b**) Reproductive number $R_t$. The case and instantaneous reproductive numbers are blue and magenta lines. The black dashed lines show the dates when each intervention policy was implemented. The red dashed line is $R_t=1$. (**c**) Infection proportions at each contact place per intervention policy level. Colors indicate different places; dark grey, red, green, grey, blue, and light grey represent households, school classrooms, workplaces, religious facilities, meetings with friends, and random encounters, respectively (top $\rightarrow$ bottom).

Using the IBM simulation results with $\beta _n = 0.8$, $\left<\rho \right>=1$, and $\sigma _\rho =0.5$, we evaluate the effectiveness of the intervention policy. Figure 7a is the incidence of infection plotted on a map of 250 regions (Log-Scale). The date of each map is the midpoint of each phase (level) of the intervention policy. The reproductive number $R_t$ is the parameter that numerically represents the spread of infection over time. $R_t$ is defined as the average number of secondary cases per primary case. We calculate $R_t$ and examine how $R_t$ changes when the intervention policy is strengthened (see, Fig. 7b). Among the various ways to calculate $R_t$, we use two methods; case reproductive number $R_t^{\rm{case}}$ and instantaneous reproductive number $R_t^{\rm{inst}}$. The advantage of the IBM simulation is that the infector and infectee can be tracked exactly. The feature allows us to compute $R_t^{\rm{case}}$ exactly. $R_t^{\rm{case}}$ is the average number of individuals infected by those who were infected at *t* day^9^. The estimation of $R_t^{\rm{case}}$ is right censored because we need to incident data later than *t* day. For more responsive to intervention policies than $R_t^{\rm{case}}$, so we calculate $R_t^{\rm{inst}}$. $R_t^{\rm{inst}}$ is the number of new infected individuals at *t* day divided by the sum of the infectiousness $\varphi$ of the infector that can infect others at *t* day^10^. On December 24, 2020, $R_t^{\rm{inst}}$ becomes less than 1. It means that COVID-19 is no longer spreading. It is right after level-2 was implemented. $R_t^{\rm{inst}}$ decreases response to the intervention policy implemented.

Previously, we numerically exhibited that the spread of disease decreased as the intervention policy was gradually tightened. We investigate how the infection proportion of contact places changes at each intervention policy level. We get the infection proportion in 6 contact places *n*. If an infection occurs at a place, we count 1 for the place. If the new infected individual had come into contact with infectors multiple places on the day of infection, we assign $1/n'$ to each place ($n'$ is the total number of places where the new infected individual had come into contact with the infectors). In Fig. 7c, level-0 is the infection proportion per place during no intervention policy. Most infections occur in the household, followed by the workplace, random encounters, and meetings with friends. Level-1 is when intervention policy was implemented for schools and workplaces. Compared to level-0, the infection proportion in schools and workplaces decreases. Level-2 is when the ban on gatherings of more than 5 individuals in the national capital area was implemented in addition to level-1. The infection proportion in friends’ meetings decreases. Level-3 is when level-2 was expanded nationally. Compared to other periods, the infection proportion in friend gatherings reduces significantly. As a result, when the intervention policy is implemented, the incidence of infection decreases in the contact places affected by the intervention policy.

### Assuming non-pharmaceutical interventions for January 2022

**Figure 8:**
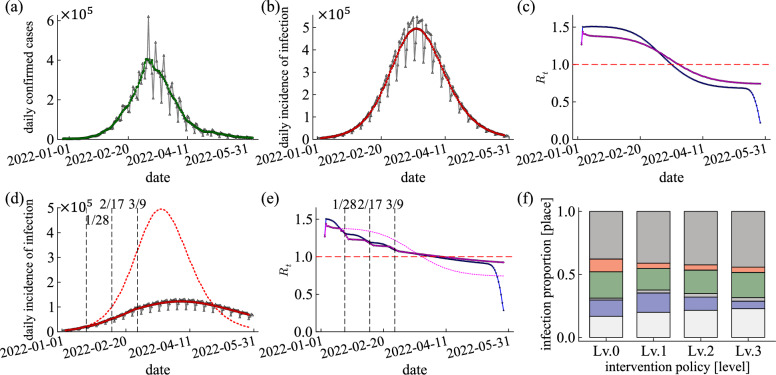
(**a–c**) Results without the intervention policies. (**d–f**) Results with the intervention policies (if the NPIs were implemented). (**a**) Actual time series of new confirmed cases in South Korea for 150 days from January 9 to June 7, 2022. The grey line is daily confirmed cases. The green line results from a 7-day moving average on the daily confirmed cases. (**b**) Simulation results for daily incidence of infection with $\beta _n = 1.05$, $\left<\rho \right>=1$, and $\sigma _\rho = 0.5$ (CI 50%). The grey line is the daily incidence of infection. The red line results from a 7-day moving average on the daily incidence of infection. (**c**) Reproductive number $R_t$. The case and instantaneous reproductive numbers are blue and magenta lines. The red dashed line is $R_t=1$. (**d–f**) Simulation results for the what-if situation with $\beta _n = 1.05$, $\left<\rho \right>=1$, and $\sigma _\rho = 0.5$. (**d**) Simulation results for daily incidence of infection. The red line is the what-if simulation results (CI 50%). The red dotted line is the daily incidences of infection without the intervention policies (red line in (**b**)). The dashed lines show the dates when each intervention policy was implemented. (**e**) Reproductive number $R_t$. The case and instantaneous reproductive numbers are blue and magenta lines. The magenta dotted line is the instantaneous reproductive number without the intervention policies (magenta line in (**c**)). The red dashed line is $R_t=1$. (**f**) Infection proportion at each contact place per the intervention policy level. Colors indicate different places; dark grey, red, green, grey, blue, and light grey represent households, school classrooms, workplaces, religious facilities, meetings with friends, and random encounters, respectively (top $\rightarrow$ bottom).

In January 2022, COVID-19 rapidly spread in South Korea. However, strong intervention policies were not implemented during the period. We estimate how much the infection would have been reduced if the intervention policies had been implemented. First, we reproduce the actual situation without intervention policies to analyze how much infection spread is reduced by the intervention policies described above. We simulate 150 days from January 9, to June 7, 2022. We assign initial immune and exposed individuals in the synthetic population to simulate. We use the line-level case data provided by GFID. Similar to the method of initial setting in November 2020, we sort the confirmed cases through January 8 by region and age group and randomly assign *R* in the synthetic population based on the population of each condition. We classify confirmed cases for 5 days from January 4 to 8 by region and age group and randomly allocate *E* in the synthetic population to simulation on January 9, 2022. Figure 8a shows the actual daily new confirmed cases for 150 days. Figure 8b represents the daily incidence of infection, the simulation results of reproducing Fig. 8a. The total number of simulations is $50+$. The infected population increased because intervention policies weren’t implemented during the period. Figure 8c shows $R_t^{\rm{case}}$ and $R_t^{\rm{inst}}$. The sensitivity analysis of the parameter is shown in Fig. S10 in the Supplementary Material.

If the intervention policy had been implemented in January 2022, We would expect infection spread is decreased. To demonstrate the expectation, we apply the three-phase intervention policies implemented from November 2020 to January 2021 to the period (150 days from January 9 to June 7, 2022). The implemented date of each intervention policy is arbitrarily assumed. The three intervention policies is implemented on January 28, February 17, and March 9, 2022. Figure 8d shows the daily incidence of infection. Although the infected population is not immediately decreased as clearly as in 2020 because there are already too many infectors in this period, we confirm an apparent decrease in the ratio of infection spread compared to results without intervention policies. Over the 150 days, the infected population is reduced by about 40.4% compared to results without intervention policy, and the peak is delayed. In the absence of intervention policies, the daily infections peak 82 days after the start of the simulation. With intervention policies, the daily infections peak 98 days after the start of the simulation. The decrease can also be validated numerically using $R_t$ (see, Fig. 8e). When the intervention policy is implemented, $R_t^{\rm{inst}}$ decreases immediately. When comparing $R_t^{\rm{inst}}$ with and without intervention policies, it is clear that $R_t^{\rm{inst}}$ with intervention policies is smaller. Although the three-phase intervention policies cannot completely eliminate the spread of disease, the infection proportion at target places reduces (see, Fig. 8f). From these results, we can conclude that the spread of COVID-19 could have been arrested in January 2022 if a valid intervention policy had been implemented (see, Fig. S12 in the Supplementary Material for a sensitivity analysis of the parameters).

## Conclusion

We tried to quantitatively derive the effect of South Korea’s COVID-19 intervention policies. In order to evaluate the effectiveness of the intervention policy, we developed the IBM simulation that uses the synthetic population with the same size and sociodemographic characteristics as South Korea. In our IBM simulation, the individuals spend a daily routine (visits various places) according to each demographic attribute. The individuals are infected stochastically due to contact with infectors daily. We analyzed the three-phase intervention policy implemented from November 2020 to February 2021 in South Korea. The infection spread quickly during this period, and intervention policies were gradually strengthened three times. On December 8, 2020, the first policy was implemented to reduce the population density of schools and large workplaces by 1/3 (2/3 for high schools, Level-1). We reproduced infection spread and intervention policies of the period. The two reproductive numbers and the daily incidence of infection were measured to assess the effectiveness of intervention policies. If the policies were implemented at a different pandemic, we also predicted how the spread of infection would have changed. The period was from January to June 2022, when the new confirmed cases increased. We reproduced the actual situation in which the policy was not implemented. Then, the spread of infection was computed if the three-phase intervention policy was implemented. We derived the results that the daily incidence of infection decreased by about 40% depending on whether the intervention policies were implemented. As a result, even in January 2022, when the confirmed cases soared, we showed that if strong intervention policies were implemented, the spread of infection could be sufficiently mitigated.

## Discussion

IBMs are highly complex as they consider all of an individual’s contacts. The more closely IBMs approximate reality, the more parameters are demanded. IBMs also have significant variances depending on the initial states. Some IBMs are stochastic simulations, which require multiple runs to see the distribution of results, which take more computing time than deterministic models. IBMs are unsuitable for rapid response to emerging infectious diseases. Nevertheless, IBMs have recently emerged as a valuable tool for predicting the spread of infectious diseases and assessing the effectiveness of intervention policies.

In this study, we developed an IBM specifically designed to replicate the implementation of intervention policies during the COVID-19 pandemic in South Korea and evaluated their effectiveness. To recapitulate the simulation results, the intervention policies slowed down and mitigated the transmission of COVID-19 in South Korea by an average 40%.

Our IBM has distinct advantages among the many IBMs that have built on statistical data. First, we made the synthetic population the same size as the population of South Korea using the IPU algorithm, which matches the household- and person-level joint distributions. Our synthetic population has about 51 million individuals. It is rare for an IBM simulation to follow the size of an actual population because the size of the synthetic population is closely related to the simulation time. This feature of our IBM allows for directly comparing simulation results with actual data. Second, we also focused on the transmission of infection among friends. Many IBM simulations using synthetic populations with sociodemographic characteristics have been developed. However, there are fewer models in which infections can spread in a community of friends by creating a friends network in the synthetic population. By endowing our synthetic population with the friend attribute, we simulated a particular intervention policy to limit the size of meetings with friends. Finally, our IBM is used line-level case data to set the initial populations of the simulation. We sorted the data by region-based age group and utilized it in the simulation.

Although our simulation results about the age group ratio and the age group-region ratio of the infected population match the actual data in this study, the simulation results for the epidemic curve don’t exactly follow the magnitude of the actual data. Our IBM wasn’t consider the possibility that infected individuals may not be diagnosed due to asymptomatic and mild symptoms. Even if an intervention policy is implemented in the real world, there is a delay between policy implementation and response time. The simulation has no delay between the two moments. The effect of the intervention policy is immediately reflected in the simulation results.

The future research may expand our IBM. We have categorized infectious disease progression into *SEIR* states, but we plan to refine it further and add states such as vaccination, asymptomatic, mild, severe symptoms, hospitalization, admissions to ICU, birth, and death. We have only analyzed Korean intervention policies, but we can use our IBM to reproduce policies such as lockdowns and social bubbles, examples of intervention policies abroad. We plan to model these various intervention policies and evaluate their effects. We will develop a more realistic respiratory infectious disease model using contact patterns obtained from the population-based survey and wireless sensor technology results^35,36^. Especially if contact patterns by age and region in South Korea are available, our IBM may better reflect reality. We show the sensitivity analysis to fit the simulation result to actual data in this study, but we do not perform the parameter estimation with a confidence interval. It can provide more accurate parameter values than sensitivity analysis. Our future work will include the parameter estimation with a confidence interval. Finally, our IBM can be a base model for studying the economic impact of the pandemic or the socioeconomic effects of intervention policies.

### Supplementary Information

Supplementary Information.

## Data Availability

The simulation code is available in https://github.com/MkChae/IBMGitHub of M.-K.C.. All input data for the simulation code are available in https://drive.google.com/drive/folders/1P-xZ2HigkeVth7_Ofw_KuP2o2kXNpk6l?usp=sharingGoogle Drive of M.-K.C. Computing resources: The calculation takes on an Intel Xeon Processor E5-2667 v4 (2019H110004, NFEC-2016-10-212540) and Intel Xeon Platinum 8358 Processor (NFEC-2023-03-285723) PC running Linux in NIMS.
