# Supplementary material for: Evaluation of COVID-19 intervention policies in South Korea using the stochastic individual-based model
Source: Sci Rep. 2023 Nov 2;13:18945. doi: 10.1038/s41598-023-46277-8 (PMC10622523; doi:10.1038/s41598-023-46277-8)
Supplement: Supplementary file 1 — Supplementary Information. [file 41598_2023_46277_MOESM1_ESM.pdf]

Supplementary Material

Contents

1 The synthetic population with sociodemographic characteristics 2
1.1 Household . . . 2
1.2 Residence region . . . 2
1.3 Age . . . 2
1.4 Education . . . 2
1.5 Economy . . . 3
1.6 Religion . . . 5
1.7 Friends . . . 5

2 Non-Pharmaceutical interventions 6
2.1 Reducing school attendance crowding . . . 7
2.2 Reducing density in workplaces . . . 7
2.3 Limit the size of private meetings . . . 8

3 Simulation results 9
3.1 November 2020 with the intervention policy . . . 9
3.2 January 2022 without the intervention policy . . . 10
3.3 January 2022 with the intervention policy . . . 11

1 The synthetic population with sociodemographic characteristics

The synthetic population is assigned sociodemographic characteristics to match statistical data of South Korea.

1.1 Household

In the synthetic population, household information is the household ID belonging to an individual. Household is information from the 2% census. Individuals (family) living in the same household have the same household ID (number). The household has only one household ID. The household ID is national level. The number of households in South Korea is 21,448,463, and the number of households in the census is 382,217. The number of households in the synthetic population is 21,471,466.

1.2 Residence region

Individuals in the synthetic population have geographic information. The country is divided into 250 regions in the simulation. The region is identified by a number (1-250). The geographic information included in the census is the residence region. Individuals of the same household ID have the same residence region number.

1.3 Age

Individuals in the synthetic population have age information. The census contains age information. In the census, the age is between 0 and 85. The synthetic population generated by expanding the census also only has 0-85 years old individuals. South Korea's 85+ population corresponds to 85 in the synthetic population. The average age of South Korea is 43.9 years old, and the average age of the census is 42.0 years old. The average age of the synthetic population is 42.9 years old.

1.4 Education

The synthetic population has an individual's education information. Education information indicates whether an individual is currently enrolled in an educational institution. Individuals attending the educational institution are students and teachers. They have the school classroom number they belong to as their education attribute. Students' ages range from 3 to 18 years old. Teachers range in age from 19 to 84. In the simulation, we assume that there are four types of educational institutions. They are kindergarten, elementary school, junior school, and high school. Students can enroll in an age-appropriate educational institution. Kindergarten is available for ages 3 to 6. The age ranges for elementary, junior, and high school are 7-12, 13-15, and 16-18, respectively.

We make a student in the synthetic population. Students in the synthetic population belong to the school classroom like students in the real world. Each school classroom is identified by a number. These classroom numbers are national level, like household IDs, but are separated by the educational institutions. To create a student in the synthetic population, we do the following. (1) We randomly select an individual to be a student. (2) We give the selected individual a classroom number. Each step is described in detail below. First, we calculate the number of students needed for each region. The number of students per region in the synthetic population is calculated using the regional age-specific enrollment ratio by KOSIS for each institution. Second, we select as many individuals in the age range from the synthetic population as students calculated for each region in the previous step. These selected individuals are students from now on. Finally, these students should have the classroom number. We assign classroom numbers to the students in the synthetic population based on the average classroom size per region by the educational institution. As a result, classmates are the same age and live in the same residence region. The total number of students in the synthetic population is 5,935,739, and the total number of classrooms is 270,104.

For example, according to KOSIS, the 2021 kindergarten enrollment ratio in Seoul is 31%, with an average classroom size of kindergarten 18.89 in Seoul. (1) The number of Seoul 3-year-olds in the synthetic population is 52,638. We randomly select about 16,319 of them and set 16,319 as students

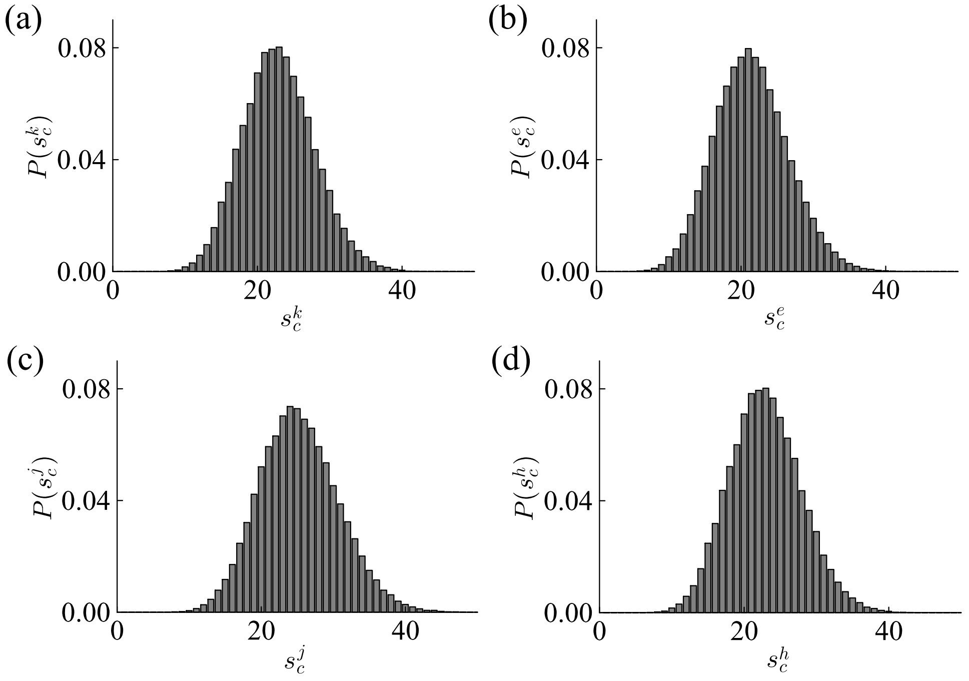

Figure S1: School classroom size s_c distribution by educational institutions (only students). (a) Distribution of kindergarten classroom size with a mean of 17.201 and a standard deviation of 4.477. (b) Elementary school classroom size distribution with a mean of 21.426 and a standard deviation of 5.076. (c) Junior school classroom size distribution with a mean of 25.255 and a standard deviation of 5.498. (d) High school classroom size distribution with a mean of 17.201 and a standard deviation of 4.971.

($52638 \times 0.31 \cong 16319$). (2) In this case, the number of classrooms needed in Seoul is 864 ($16319/18.89 \cong 864$). The selected Seoul 3-year-old students are randomly assign a number from 1 to 864. It is the classroom number. In the simulation, we subdivide Seoul and calculate the entire country into 250 regions.

We make teachers in the synthetic population. Each classroom has teachers. Teachers are selected from the economically active population (see, Section 1.5). The number of teachers needed for each classroom is dependent on the data of students per teacher by each educational institution. According to e-Narajiphyo data, the 2022 average students number per teacher in elementary schools is 13.7, and in middle schools is 11.7.

For example, if a classroom in an elementary school has 38 students, the teacher allocates 3 seats ($38/13.7 \cong 3$).

Reference: [1, 2, 3, 4, 5, 6]

1.5 Economy

The synthetic population contains economy information. Economy information indicates whether an individual is economically active or not. The age of the economically active population is limited to 19-84 years old. They can commute to other areas where they don't live. In other words, an economically active individual's residence and working region may be different. They have their working region in their economic attribute. There are two kinds of employees; office workers and teachers. Office workers have a workplace number in their economy information. Teachers have a school classroom number that corresponds to their workplace number.

Here, we set up the economically active individuals in the synthetic population. Employees in the synthetic population, like employees in the real world, have information about the workplace (school classroom) and the working region where the workplace (classroom) is located. Each workplace is represented by a number, which is the national level. The working region is represented by a number (1-250). To set up employees in the synthetic population, we can do the following. (1) We randomly

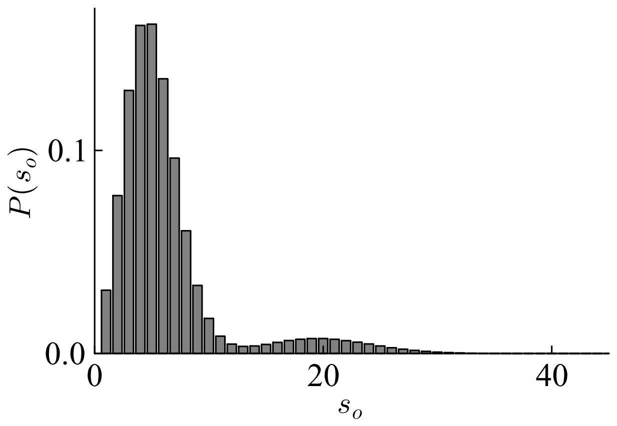

Figure S2: Workplace size s_o distribution with a mean of 6.278 and a standard deviation of 4.814.

select an individual to be economically active. We assign the selected individual a (2) working region and (3) workplace (classroom) number. This sequence is described in detail as follows. First, we calculate the number of economically active people needed in each region. In the synthetic population, the economically active population by region is allocated using employment ratio data by region and age group from KOSIS. Second, an employee can commute to a region other than the residence region in the simulation. We calculate the inter-regional commuting ratio based on commuting data from the Korea Telecom DataBase (KT DB) and use it to give the synthetic population their working regions. The data is sorted into regions by age group. Third, we calculate the number of teachers needed in each region. We randomly select the required number of teachers in employees who commute to this region. The selected individual is set as a teacher. These teachers are given classroom numbers. Finally, employees, except for teachers, have a workplace number. The method for assigning workplace numbers is the same as for assigning classroom numbers. We get the number of workplaces needed for each region and assign workplaces numbers to office workers. However, we assume two workplace sizes unlike educational institutions; small and large workplace. The average size of each workplace is 5 and 20. The distribution of workplace size is a double-gaussian. The proportion of large and small workplace is assumed based on the number of businesses by region and employee size from KOSIS. The workplace number is the national level, but it does not distinguish between large and small workplace. As a result, the number of economically active individuals in the synthetic population is 28,236,447, and the total number of offices is 4,417,708. The average of workplace size is 6.278, and a standard deviation is 4.814.

For example, (1) according to employment ratio data in 2022 by KOSIS, the employment rate for 25-29 year olds in Seoul is 73.4%. The 25-29 years old population of Seoul in the synthetic population is 832,932. So, 611,372 of them are employees ($832932 \times 0.734 \cong 611372$). (2) According to 2017 KT DB's commuting data, the ratio of people aged 25-29 in Incheon who commute to Seoul is 9.61%. If we calculate all other regions in this way, the number of economically active people aged 25-29 who work in Seoul is 672,183. (Additionally, of the 25-29 year olds living in Seoul, 576,708 work in Seoul, and 34,664 commute to other regions.) (3) The total number of office workers in Seoul is 5,334,373 (except for teachers). According to KOSIS's 2019 data on the number of businesses by region and employee size, the ratio of small workplaces to large workplaces in Seoul is 10.6:1. The number of offices needed in Seoul is 774,580 and 73,074, respectively ($10.6 : 1 = x : y,\ 20x + 5y = 5334373 \rightarrow x = 73074,\ y = 744580$). The 3,872,870 office workers in Seoul are randomly assigned 1-774,580 (small workplace). The other office workers are randomly assigned 774,581-847,654 (large workplace). These are the workplace numbers. For the simulation, we subdivide Seoul and calculate the country into 250 regions.

Reference: [7, 8, 9]

1.6 Religion

The synthetic population contains religion information. Religion information indicates whether an individual is religiously active or not. The religiously active population is not age-restricted. The religiously active population, or religious people, have the religious facility number they attend as their religious attribute. In the simulation, we assume that there are three types of religion. They are Christianity, Catholicism, and Buddhism. They can only attend their religious facilities in residence region.

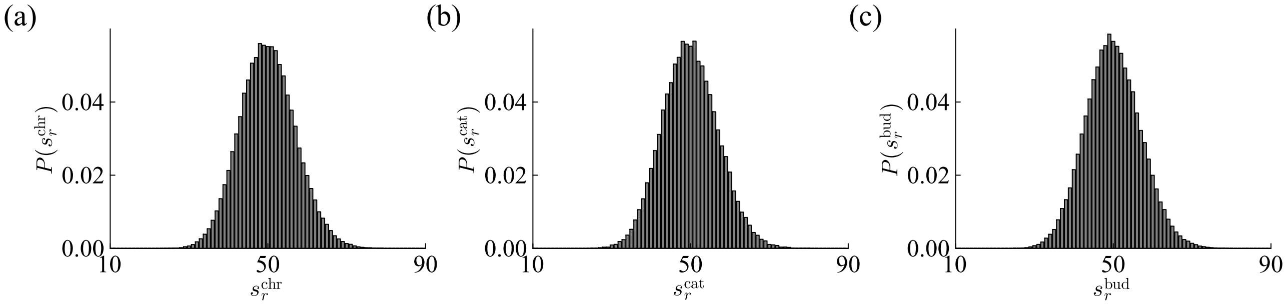

Figure S3: Religious facility size s_r distribution by religion. (a) The Christianity facility size distribution has a mean of 49.97 and a standard deviation of 7.068. (b) The Catholicism facility size distribution has a mean of 49.926 and a standard deviation of 7.071. (c) The Buddhism facility size distribution has a mean of 49.963 and a standard deviation of 7.038.

We make religious individuals in synthetic populations. Religious individuals in the synthetic population attend religious facilities, like religious people in the real world. Each religious facility is identified by a number. These numbers are national level, like household IDs, but they are separated by religion. To create religious individuals in synthetic population, we follow these steps. First, we calculate the number of religious people needed for each region. It is calculated using the ratio of religious people provided by KOSIS. Second, we randomly select individuals to become religious. We select individuals in the synthetic population per each region equal to the number of calculated religious people. These selected individuals are now religious. Third, we determine the religion that the religious individual believes. It is computed using the ratio of adherents per religion provided by KOSIS. We assign a religion in each region. Finally, we give them religious facility numbers to attend. These religious individuals have the religious facility number. In the simulation, we assume that the average size of a religious facility is 50. The method for assigning religious facility numbers is the same as assigning school classrooms. We calculate the number of religious facilities needed and randomly assign a religious facility number to each religious individual. As a result, religious individuals who attend the same religious facility live in the same residence region. In the synthetic population, the number of religious individual is 10,388,811, 4,174,982, and 8,179,924, in order of Christianity, Catholicism, and Buddhism, and the number of religious facilities is 207,899, 83,623, and 163,718.

For example, according to 2015 KOSIS data, the religious people ratio is 43.9%. (1) The population of Seoul is 9,472,840 in the synthetic population. 4,158,577 of them are religious ($9472840 \times 0.439 \cong 4158577$). (2) According to KOSIS data, the ratio of adherents of Christianity, Catholicism, and Buddhism is 45.7%, 18.3%, and 36.0%. The number of adherents of each religion in Seoul is 1,900,470, 761,019, and 1,497,088 ($4158577 \times [0.457, 0.183, 0.360] \cong [1900470, 761019, 1497088]$). (3) The number of religious facilities needed in Seoul is 38,009, 15,220, and 29,942, respectively ($[1900470, 761019, 1497088]/50 \cong [38009, 15220, 29942]$). Each religious adherent in Seoul is randomly assigned a number (1-38,009, 1-15,220, and 1-29,942, respectively). These numbers represent religious facilities they attend. For the simulation, we subdivide Seoul and calculate the country into 250 regions.

Reference: [10]

1.7 Friends

The synthetic population contains friend information. For each individual, the friend information is a list of their friends' person IDs. A person ID is a individual's index number. The friend list is a

bidirectional relationship. If ‘A’ has ‘B’ in A’s friend list, ‘B’ also has ‘A’ in B’s friend list. Since this simulation focuses on the spreading of local infections, only residents can be friends. Also, individuals under the age of 2 have no friends.

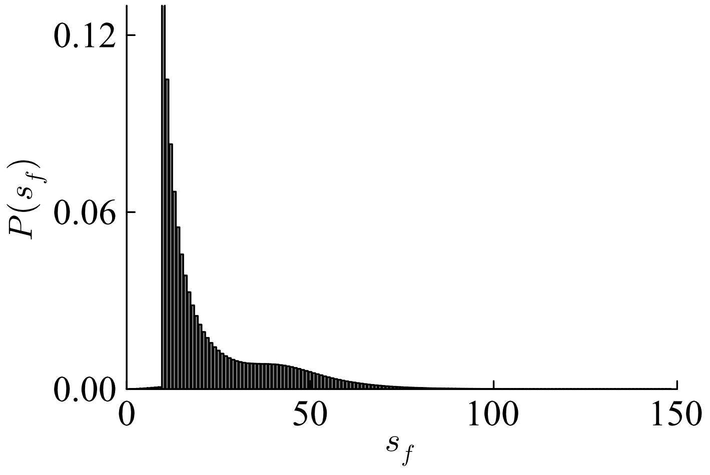

Figure S4: The number of friends s_f distribution with a mean of 22.2 and a standard deviation of 15.0

The list of friends in the synthetic population is generated by a Barabasi-Albert network, a scale-free network [11]. The link in the network is bidirectional. The network generation methods for (1) adults (ages 19+) and (2) minor (ages 3-18) are separate. (1) All the friends of adults are adults. Individuals are grouped together to growth an adult’s friend network. The adults are divided into 200 randomly selected individuals of the same age group, with 10 years intervals, and live in the same residence region. Each group of 200 individual is grown with the Barabasi-Albert algorithm. In this network, connected individuals is set as friends of each other. They write each other’s person ID in their friend lists. We use the following parameters to generate the Barabasi-Albert network. The initial network consists of 15 nodes (vertices) connected by a cycle. In the initial network, we add one node at every step, which connects 10 edges (links) to existing nodes. It completes the list of friends of the adults using the Barabasi-Albert network. (2) The friends of the minors are all minors. The friends of minors are created in three steps. (i) There is the same way like adults. The only difference is the age condition. Group 200 minors who belong to the same age group attend the same educational institutions and live in the same residence region. Each minor group is grown with the Barabasi-Albert algorithm. The parameters are the same as adults. We also created (ii) classmates and (iii) schoolmates for students enrolled in educational institutions. (ii) For classmates, each classroom network is grown with the Barabashi-Albert algoritm. The network parameters are as follows. The initial network is a randomized connection of half the number of students in the classroom n_c with 90% edges. We add one node for every step, which connects $n_c/2$ edges to other nodes. (iii) For school friends, we group 10 classrooms with the same age and the same residence region. Each school network is grown with the Barabasi-Albert algorithm. The network parameters are the same as when creating classmates. However, the total size is the number of students in the school n_s.

In the simulation, the average number of friends is 22.2 and a standard deviation is 15.0 for the synthetic population (ages 3+). The individual with the most friends has 148 friends, and and the fewest has 2 friends. Students have more friends because they have been connected three times.

Reference: [11]

2 Non-Pharmaceutical interventions

We have reproduced the three-phase intervention policies implemented from November 2020 to February 2021 in the text. During this time, the intervention policy was tightened three times. On December

8, 2020, the first policy was implemented to reduce the population density of schools and large workplaces by 1/3 (2/3 for high schools, Level-1). On December 24, 2020, the friends' meeting size was additionally limited to 4 people or less in the national capital area (Level-2). On January 4, 2021, the policy of limiting the friends' meeting size was expanded nationwide (Level-3). In the supplementary, we describe each intervention policy in more detail.

2.1 Reducing school attendance crowding

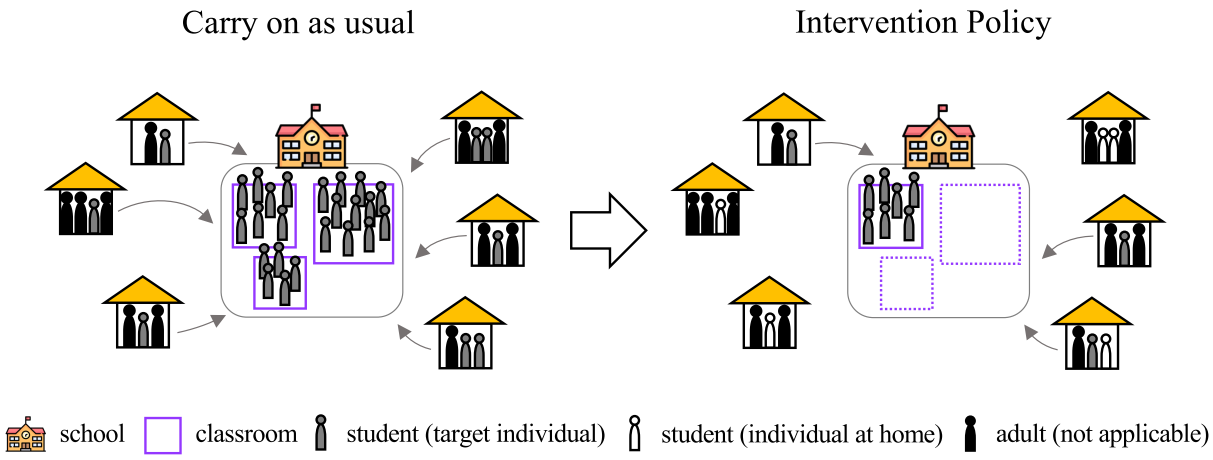

Figure S5: Schematic diagram of the intervention policy that reduces attendance density in schools by one-third. Purple squares represent classrooms. Purple squares with a dashed line are classrooms that do not attend school. Grey individuals (students) are targets in this policy. White individuals are also targets, which are students staying home due to this policy. Black individuals are unaffected by this policy, which means adults.

When level-1 was implemented, the intervention policy for the schools we reproduced was as follows: Authorities advised schools to reduce attendance density. Students who used to go to school every weekday attend classes remotely from home when intervention policies are in place. Only 1/3 of the total classrooms could be allowed to attend school. The number of attending classrooms should be at most 1/3 of the total number of classrooms. The number of students in each classroom does not matter. On school days, students go to school as usual. However, on non-school days, students are educated at home through homeschooling. Teachers go to work as usual each weekday. In South Korea, each grade (age) went to school. The exception was for high school, where up to 2/3 of the class could attend.

Figure S5 shows the intervention policy for the schools. Figure S5 (left) is a schematic diagram of a state without the intervention policy. Students go to school every weekday, and each has a classroom to which they belong. Figure S5 (right) is a schematic diagram of a state with reduced attendance density in schools. Only 1/3 of the total class can go to school. Students in classrooms that do not go to school today stay home. Tomorrow, students in other classrooms go to school.

2.2 Reducing density in workplaces

When level-1 was implemented, the intervention policy for the workplace we reproduced was as follows: Authorities advised workplaces to reduce population density. Office workers who used to go to workplaces every weekday work remotely from home when intervention policies are in place. In South Korea, only up to 1/3 of the workplace's capacity could work in the workplace. The remaining office workers had to work in the home.

Figure S6 shows the intervention policy for the workplace. Figure S6 (left) is a schematic diagram of a state without the intervention policy. Office workers come to work every weekday. Figure S6 (right) is a schematic diagram of a state with reduced crowding in workplaces. Only a third of the workplace's population is allowed to come to the workplace. The rest of the office workers do telework.

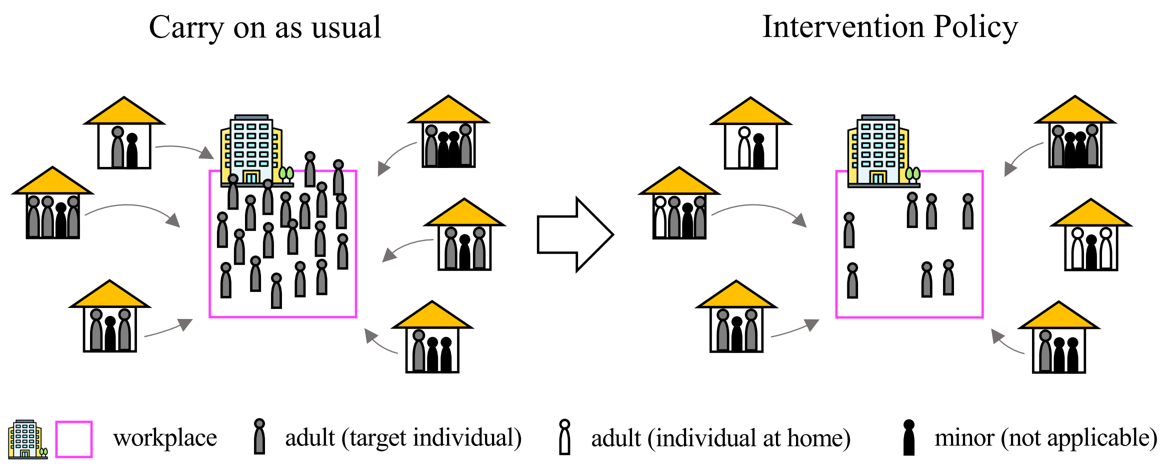

Figure S6: Schematic diagram of the intervention policy that reduces crowding in workplaces by one-third. Magenta square represents workplaces. Grey individuals (office workers) are targets in this policy. White individuals are also targets, which are office workers staying home due to this policy (telework). Black individuals are unaffected by this policy, which means minors (children).

Tomorrow, another individual will work in the workplace. In the simulation, individuals who will work at the workplaces are randomly selected each weekday.

2.3 Limit the size of private meetings

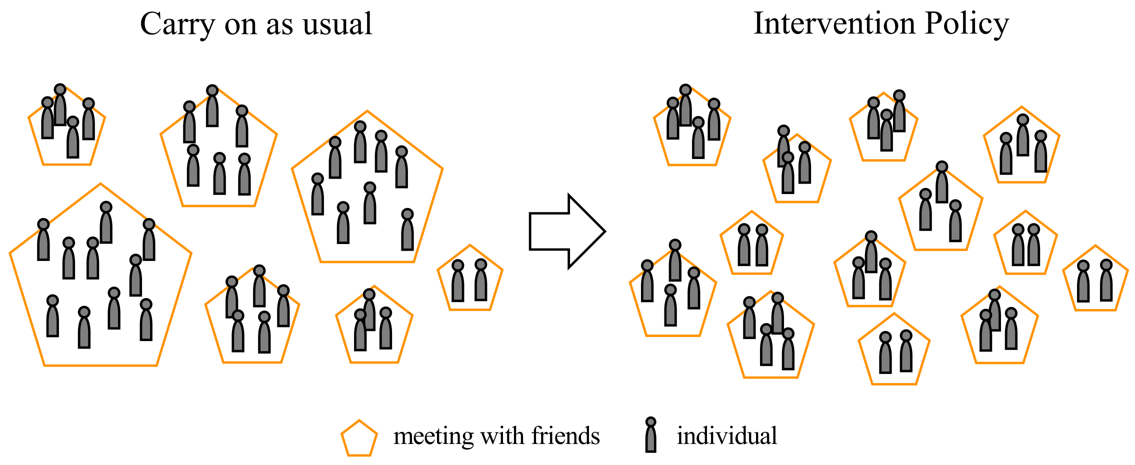

Figure S7: Schematic diagram of limiting the friends' meeting size (intervention policy). Orange pentagons represent meetings with friends.

When level-2 and -3 were implemented, authorities advised people to keep the size of private gatherings to four people or less. During this period, the size of meetings with friends was limited. The intervention policy we reproduced was restricting the size of meetings with friends. Individuals are allowed friend groups to be no larger than four people. Initially, this policy was only implemented in the national capital area (Seoul, Incheon, and Gyeonggi, level-2) but was soon expanded to the entire country (level-3).

Figure S7 shows the intervention policy on friends' meeting sizes. Figure S7 (left) is a schematic diagram of a state without intervention policy. However, the maximum size of a friend meeting allowed in the simulation is 10 individuals. Figure S7 (right) is a schematic diagram of a state with restrictions on the size of friend meetings. Individuals are allowed to meet with up to 3 friends at a time.

3 Simulation results

The simulation results are obtained by varying the probability of infection for the three situations we reproduced (2020, 2022, and what-if 2022). We change the transmission coefficients β_n and standard deviation of relative infectiousness σ_ρ (The mean of relative infectiousness is fixed: $\langle\rho\rangle = 1.$), which have the most significant effect on the probability of infection.

3.1 November 2020 with the intervention policy

We reproduce the 100-day intervention policies from November 1, 2020, to February 8, 2021. We compute the incidence of infection and the reproductive number.

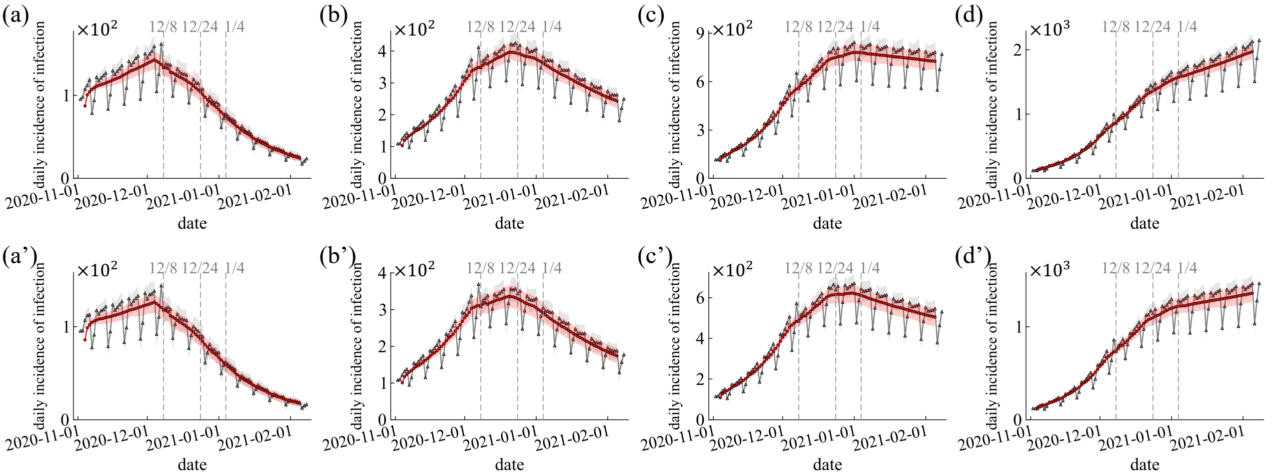

Figure S8: Simulation results for the daily incidence of infection (50% confidence interval). (a) $\beta_n = 0.7$. (b) $\beta_n = 0.8$. (c) $\beta_n = 0.85$. (d) $\beta_n = 0.9$. The top panels are the relative infectiousness ρ has no heterogeneity ($\langle\rho\rangle = 1$ and $\sigma_\rho = 0$). The bottom panels are the relative infectiousness ρ has a gamma distribution with a mean of 1 and a standard deviation of 0.5 ($\langle\rho\rangle = 1$ and $\sigma_\rho = 0.5$). (b') The main paper data. The grey line is the daily incidence of infection. The red line results from a 7-day moving average on the daily incidence of infection. The dashed lines show the dates when each intervention policy was implemented.

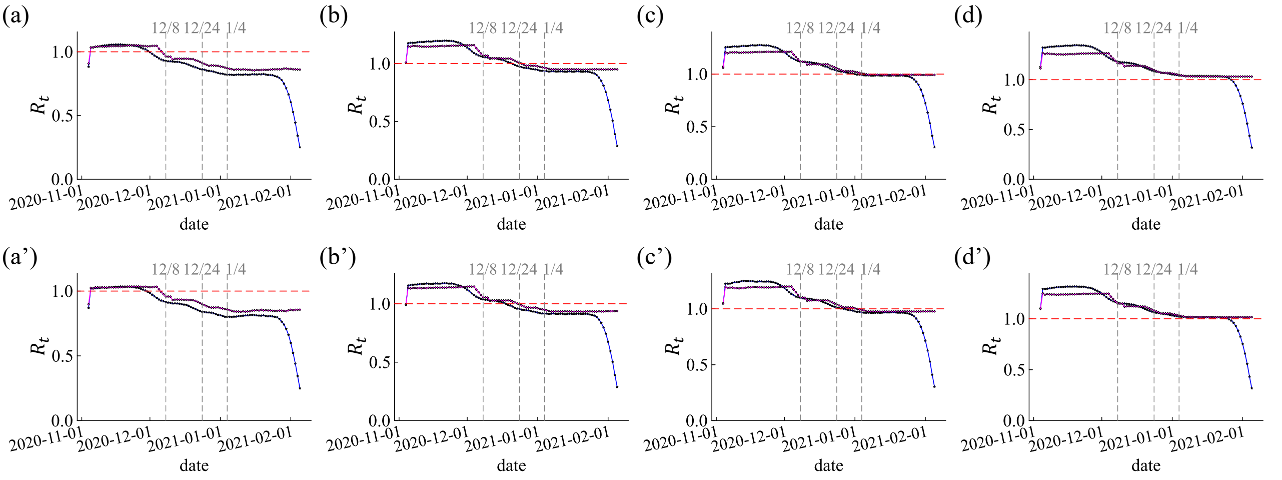

Figure S9: Simulation results for the reproductive number R_t. (a) $\beta_n = 0.7$. (b) $\beta_n = 0.8$. (c) $\beta_n = 0.85$. (d) $\beta_n = 0.9$. The top panels are the relative infectiousness ρ has no heterogeneity ($\langle\rho\rangle = 1$ and $\sigma_\rho = 0$). The bottom panels are the relative infectiousness ρ has a gamma distribution with a mean of 1 and a standard deviation of 0.5 ($\langle\rho\rangle = 1$ and $\sigma_\rho = 0.5$). (b') The main paper data. The case and instantaneous reproductive numbers are blue and magenta in color. The grey dashed lines show the dates when each intervention policy was implemented. The red dashed line is $R_t = 1$.

3.2 January 2022 without the intervention policy

We reproduce 150 days from January 9, 2022, to June 7, 2022. We compute the incidence of infection and the reproductive number.

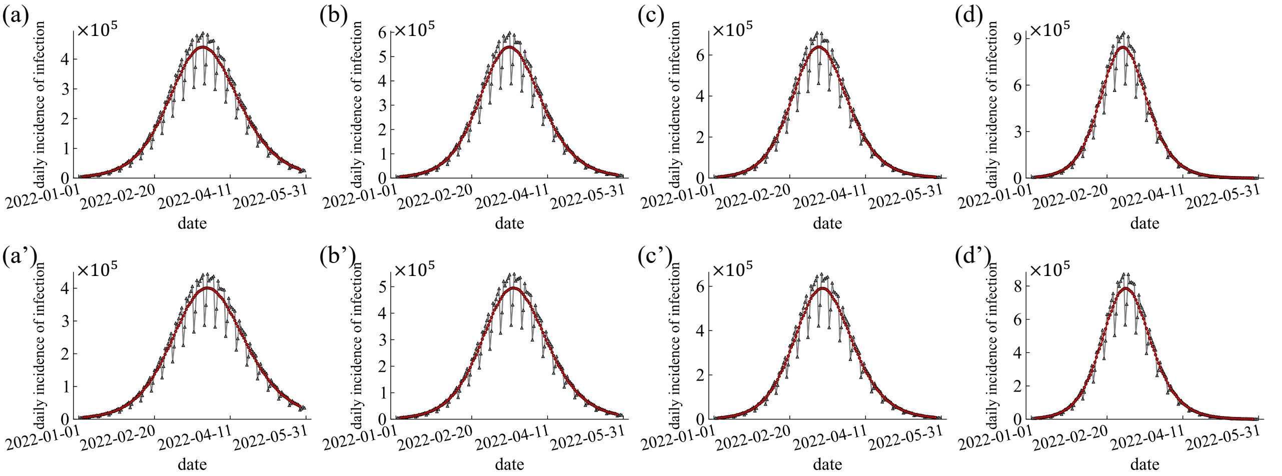

Figure S10: Simulation results for daily incidence of infection (50% confidence interval). (a) $\beta_n = 1.0$. (b) $\beta_n = 1.05$. (c) $\beta_n = 1.1$. (d) $\beta_n = 1.2$. The top panels are the relative infectiousness ρ has no heterogeneity ($\langle\rho\rangle = 1$ and $\sigma_\rho = 0$). The bottom panels are the relative infectiousness ρ has a gamma distribution with a mean of 1 and a standard deviation of 0.5 ($\langle\rho\rangle = 1$ and $\sigma_\rho = 0.5$). (b') The main paper data. The grey line is the daily incidence of infection. The red line results from a 7-day moving average on the daily incidence of infection.

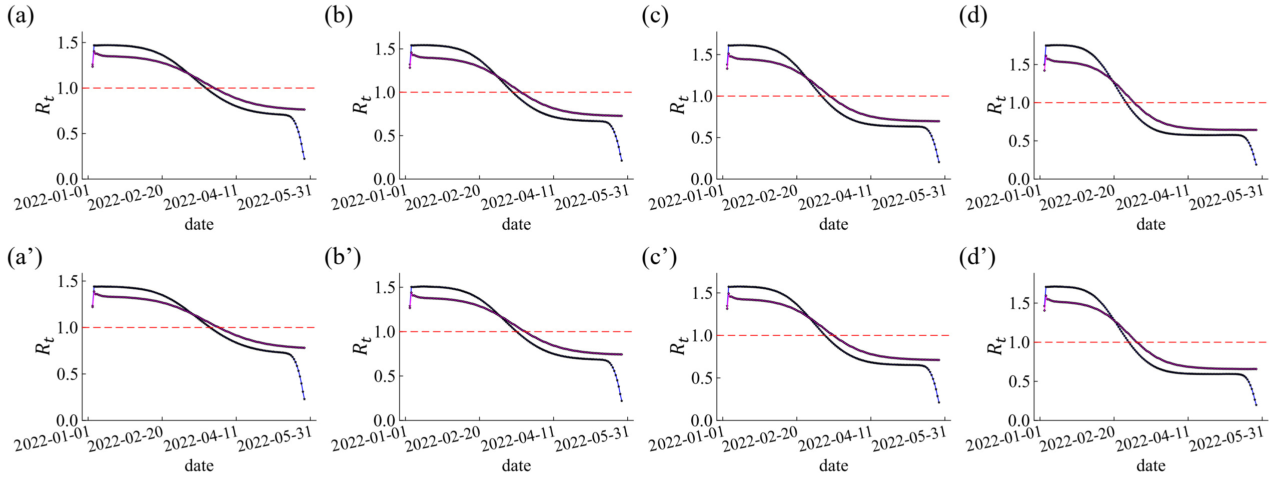

Figure S11: Simulation results for the reproductive number R_t. (a) $\beta_n = 1.0$. (b) $\beta_n = 1.05$. (c) $\beta_n = 1.1$. (d) $\beta_n = 1.2$. The top panels are the relative infectiousness ρ has no heterogeneity ($\langle \rho \rangle = 1$ and $\sigma_\rho = 0$). The bottom panels are the relative infectiousness ρ has a gamma distribution with a mean of 1 and a standard deviation of 0.5 ($\langle \rho \rangle = 1$ and $\sigma_\rho = 0.5$). (b') The main paper data. The case and instantaneous reproductive numbers are blue and magenta in color. The red dashed line is $R_t = 1$.

3.3 January 2022 with the intervention policy

We apply the three-phase intervention policies implemented from November 2020 to January 2021 to the period (150 days from January 9 to June 7, 2022). We simulate the what-if situation, and compute the incidence of infection and the reproductive number.

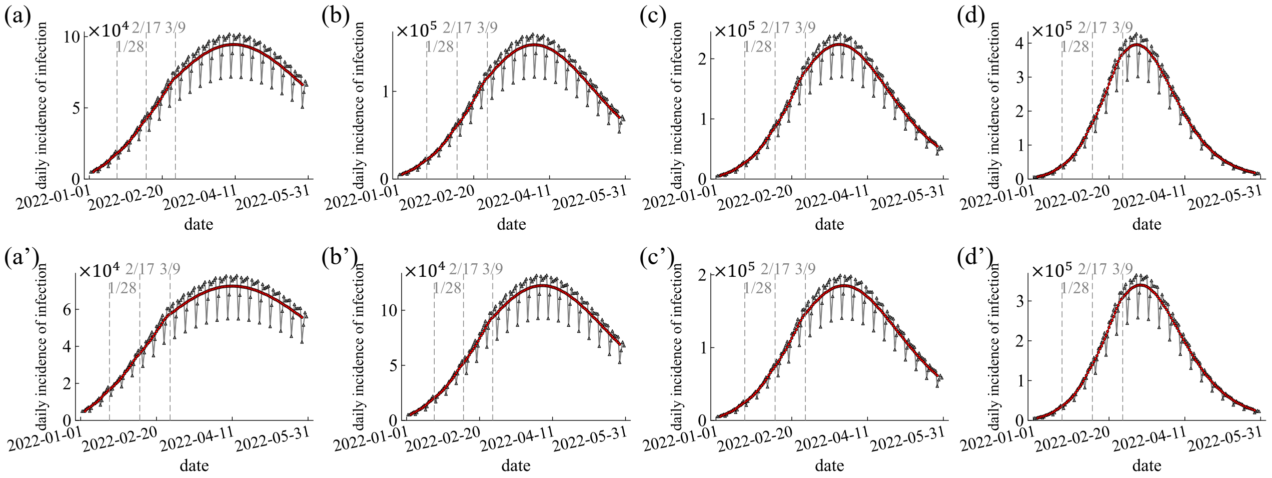

Figure S12: Simulation results for daily incidence of infection (50% confidence interval). (a) $\beta_n = 1.0$. (b) $\beta_n = 1.05$. (c) $\beta_n = 1.1$. (d) $\beta_n = 1.2$. The top panels are the relative infectiousness ρ has no heterogeneity ($\langle\rho\rangle = 1$ and $\sigma_\rho = 0$). The bottom panels are the relative infectiousness ρ has a gamma distribution with a mean of 1 and a standard deviation of 0.5 ($\langle\rho\rangle = 1$ and $\sigma_\rho = 0.5$). (b') The main paper data. The grey line is the daily incidence of infection. The red line results from a 7-day moving average on the daily incidence of infection. The grey dashed lines show the dates when each intervention policy was implemented.

References

[1] Population by age. *KOSIS* `https://kosis.kr/statHtml/statHtml.do?orgId=101&tblId=DT_1B04006&conn_path=I2`, 2022.

[2] Students per faculty member. *e-Narajiphyo* `https://www.index.go.kr/unity/potal/main/EachDtlPageDetail.do?idx_cd=1521`, 2022.

[3] Kindergarten overview. *KOSIS* `https://kosis.kr/statHtml/statHtml.do?orgId=334&tblId=DT_1963003_001&conn_path=I2`, 2021.

[4] Elementary school overview. *KOSIS* `https://kosis.kr/statHtml/statHtml.do?orgId=334&tblId=DT_1963003_002&conn_path=I2`, 2021.

[5] Junior school overview. *KOSIS* `https://kosis.kr/statHtml/statHtml.do?orgId=334&tblId=DT_1963003_003&conn_path=I2`, 2021.

[6] High school overview. *KOSIS* `https://kosis.kr/statHtml/statHtml.do?orgId=334&tblId=DT_1963003_004&conn_path=I2`, 2021.

[7] Working-age population by age. *KOSIS* `https://kosis.kr/statHtml/statHtml.do?orgId=101&tblId=DT_1DA7015S&conn_path=I2`, 2022.

[8] Number of businesses by employee size. *KOSIS* `https://kosis.kr/statHtml/statHtml.do?orgId=101&tblId=DT_1K52C03&conn_path=I2`, 2019.

[9] Airplane and train ridership: 2020-od-psn-mod-10. *KTDB* `https://www.ktdb.go.kr`, 2020.

[10] Adherents by religion. *KOSIS* `https://kosis.kr/statHtml/statHtml.do?orgId=101&tblId=DT_1PM1502&conn_path=I2`, 2015.

[11] Albert-László Barabási and Réka Albert. Emergence of scaling in random networks. *science*, 286(5439):509–512, 1999.

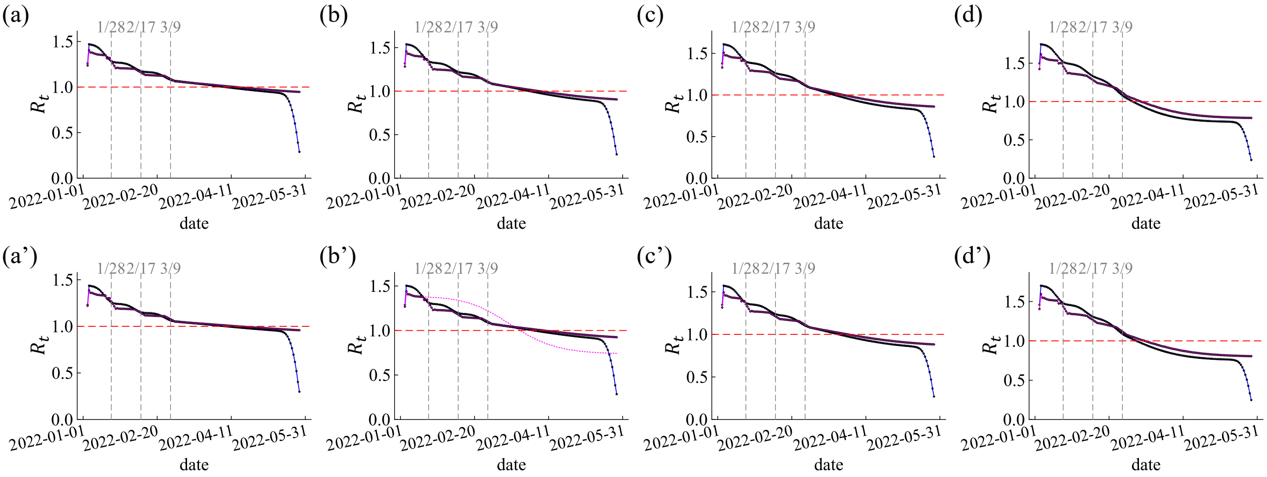

Figure S13: Simulation results for the reproductive number R_t. (a) $\beta_n = 1.0$. (b) $\beta_n = 1.05$. (c) $\beta_n = 1.1$. (d) $\beta_n = 1.2$. The top panels are the relative infectiousness ρ has no heterogeneity ($\langle\rho\rangle = 1$ and $\sigma_\rho = 0$). The bottom panels are the relative infectiousness ρ has a gamma distribution with a mean of 1 and a standard deviation of 0.5 ($\langle\rho\rangle = 1$ and $\sigma_\rho = 0.5$). (b') The main paper data. The case and instantaneous reproductive numbers are blue and magenta in color. The grey dashed lines show the dates when each intervention policy was implemented. The red dashed line is $R_t = 1$.
